# Metabolite and transcript profiling of Guinea grass (*Panicum maximum* Jacq) response to elevated [CO_2_] and temperature

**DOI:** 10.1007/s11306-019-1511-8

**Published:** 2019-03-25

**Authors:** Jessica M. Wedow, Craig R. Yendrek, Tathyana R. Mello, Silvana Creste, Carlos A. Martinez, Elizabeth A. Ainsworth

**Affiliations:** 1 Department of Plant Biology & Carl R. Woese Institute for Genomic Biology, University of Illinois at Urbana-Champaign, 1201 W. Gregory Drive, 147 ERML, Urbana, IL 61801 USA; 2 Department of Biology, FFCLRP, University of Sao Paulo, Ribeirão Preto, SP Brazil; 3 Instituto Agronômico (IAC), Centro de Cana, Ribeirão Preto, Brazil; 4 USDA Agricultural Research Service, Global Change and Photosynthesis Research Unit, Urbana, IL USA

**Keywords:** *Panicum maximum*, Metabolomics, Transcriptomics, Elevated temperature, Elevated CO_2_

## Abstract

**Introduction:**

By mid-century, global atmospheric carbon dioxide concentration ([CO_2_]) is predicted to reach 600 μmol mol^−1^ with global temperatures rising by 2 °C. Rising [CO_2_] and temperature will alter the growth and productivity of major food and forage crops across the globe. Although the impact is expected to be greatest in tropical regions, the impact of climate-change has been poorly studied in those regions.

**Objectives:**

This experiment aimed to understand the effects of elevated [CO_2_] (600 μmol mol^−1^) and warming (+ 2 °C), singly and in combination, on *Panicum maximum* Jacq. (Guinea grass) metabolite and transcript profiles.

**Methods:**

We created a de novo assembly of the *Panicum maximum* transcriptome. Leaf samples were taken at two time points in the Guinea grass growing season to analyze transcriptional and metabolite profiles in plants grown at ambient and elevated [CO_2_] and temperature, and statistical analyses were used to integrate the data.

**Results:**

Elevated temperature altered the content of amino acids and secondary metabolites. The transcriptome of Guinea grass shows a clear time point separations, with the changes in the elevated temperature and [CO_2_] combination plots.

**Conclusion:**

Field transcriptomics and metabolomics revealed that elevated temperature and [CO_2_] result in alterations in transcript and metabolite profiles associated with environmental response, secondary metabolism and stomatal function. These metabolic responses are consistent with greater growth and leaf area production under elevated temperature and [CO_2_]. These results show that tropical C_4_ grasslands may have unpredicted responses to global climate change, and that warming during a cool growing season enhances growth and alleviates stress.

**Electronic supplementary material:**

The online version of this article (10.1007/s11306-019-1511-8) contains supplementary material, which is available to authorized users.

## Introduction

Atmospheric carbon dioxide concentrations ([CO_2_]) have risen by 40% since pre-industrial times, with approximately half of that increase within the past 40 years. The International Panel on Climate Change (IPCC) has predicted that [CO_2_] will rise to 600 ppm and mean global temperature will increase 1.1 to 2.6 °C by the end of the century, with a moderate climate change projection scenario (IPCC 2014). Although the combined effects of rising atmospheric [CO_2_] and rising temperatures have been studied in field experiments in temperate forests (Sun et al. 2017; Svensson et al. 2018), croplands (Ruiz-Vera et al. 2015; Cai et al. 2016), and grasslands (Ryan et al. 2015), there have been fewer field studies of the combined effects of rising [CO_2_] and temperature in tropical and sub-tropical ecosystems. Differences in climate and nutrient limitation in tropical regions may fundamentally alter how tropical ecosystems respond to global climate change compared to well-studied temperate ecosystems (Leakey et al. 2012). Therefore, the paucity of data from tropical regions presents a significant challenge for an accurate understanding of global responses to climate change. Adding to this challenge is the realization that responses of vegetation to combined elevated [CO_2_] and temperature treatments are different from single-factor experiments, and that additive effects are rare (Dieleman et al. 2012). Thus, there is a need for multi-factor global change experiments, especially in tropical regions.

Pastures cover about 30% of the terrestrial surface in tropical regions and play an important role in carbon sequestration (Boval and Dixon 2012; Ramankutty et al. 2008). In tropical regions, C_4_ grasses dominate those pastures (Still et al. 2003). *Panicum maximum Jacq.* (Guinea grass) is a perennial C_4_ grass species native to tropical Africa, which has been adapted in Brazil as a forage and range grass (Pedreira et al. 2015). Approximately 90% of Brazilian cattle are raised on pasture land (Cardoso et al. 2016), and perennial C_4_ grasses such as Guinea grass are principle species for the Brazilian feedstock industry. Despite the economic importance of Guinea grass to this industry, very little is known about how it and other C_4_ forage grasses will respond to rising atmospheric [CO_2_] and temperature. To address this knowledge gap, a Temperature Free Air CO_2_ Enrichment (T-FACE) experiment was established in Ribeirão Preto, Brazil to expose Guinea grass to elevated [CO_2_] (600 ppm) and increased canopy temperatures of 2 °C (Britto de Assis Prado et al. 2016).

‘Omic’ technologies including transcriptomics and metabolomics provide a functional analysis to connect physiological and molecular responses to genetic and phenotypic information (Langridge and Fleury 2011). Such technologies can be used to better understand the mechanisms underpinning plant responses to global climate change and can reveal targets for improving plant performance in the future. In recent years there has been an increasing number of studies investigating transcriptomic and metabolomic responses of C_4_ grasses to a variety of abiotic stresses imposed in greenhouses and/or chambers (e.g., Sicher et al. 2012; Meyer et al. 2014; Sui et al. 2015; Toledo-Silva et al. 2013). However, there is an increasing need to investigate and understand plant transcriptional and metabolic responses to global change or abiotic stress in the field where responses can be dramatically different from controlled environments (Lovell et al. 2016). The combined use of FACE and ‘omic’ technologies provides a powerful approach for gaining new insight into plant molecular responses to global climate change in the field. Combining metabolomic and transcriptomic studies allowing for a holistic view of the plant’s response to stress.

Our study combines the power of FACE experiments for growing plants under elevated [CO_2_] and temperature in fully open-air conditions with transcriptomic and metabolomics profiling. The aims are to (1) understand the biochemical responses of Guinea grass to rising [CO_2_] and temperature when applied individually and in combination; (2) understand how gene expression is altered in each treatment; and (3) integrate the metabolomic and transcriptional responses.

## Methods

### Experimental design and tissue collection

The field experiment was conducted in a 2500 m^2^ field at the University of São Paulo in Ribeirão Preto, São Paulo Brazil. Guinea grass was planted in a completely replicated design with 4 ambient temperature and [CO_2_] plots (C), 4 elevated [CO_2_] (600 μmol mol^−1^) and ambient temperature plots (eC), 4 ambient [CO_2_] and elevated temperature [+ 2 °C] plots (eT), and 4 combined treatment (elevated [CO_2_] and elevated temperature) plots (eCeT). Each plot was 2-m in diameter; [CO_2_] was controlled using Free Air CO_2_ Enrichment, and canopy warming was provided by infrared ceramic heaters as described by Britto de Assis Prado et al. (2016). Treatment was started on April 22, 2014.

Leaf tissue was collected for transcriptomic and metabolomics analysis on two dates: May 22, 2014 (time point A—30 days of treatment), and June 14, 2014 (time point B—50 days of treatment). Six leaves per experimental plot were collected at each time point and then pooled for RNA extraction and metabolomics analysis. At the time of sampling, tissue was immediately quenched in liquid nitrogen. RNA was extracted in Brazil using the protocol described by Bilgin and colleagues (Bilgin et al. 2009), and subsequently shipped in ethanol to the U.S. for transcriptomic analysis. Lyophilized leaf tissue from the same leaves was sent for metabolite analysis. Leaves were sampled for biomass, area, and specific leaf area on August 22 and 29th, 2014 from 60 tillers per treatment as described by Britto de Assis Prado et al. (2016).

### Transcriptional analysis

RNA samples that were shipped from Brazil were re-suspended by washing with sodium acetate (pH 5.5) and glycerol. RNA quantity and quality were re-assessed using a spectrophotometer (Nanodrop 1000, Thermo Fisher) and microfluidic visualization tool (Bioanalyzer, Agilent Technologies). 32 samples (16 per time point including 4 from each [CO_2_] and temperature treatment) were submitted for library construction and sequencing at the Roy J. Carver Biotechnology Center at the University of Illinois, Urbana-Champaign. RNAseq libraries were prepared with Illumina’s TruSeq Standard RNA sample prep kit according to the manufacturer’s instruction (Illumina, San Diego, CA, USA). No public reference genome for Guinea grass was available at the time of the sequencing. Therefore, a combination of MiSeq and HiSeq was used to generate sequences of different lengths for de novo genome assembly.

For MiSeq analysis, 50 µg of total RNA per sample was pooled for library preparation without RNA fragmentation. The MiSeq library was quantified by qPCR and sequenced on one MiSeq flowcell for 301 cycles using paired-end sequencing. HiSeq paired-end sequencing was done with four quantified libraries per treatment which were pooled in equimolar concentration and sequenced on two lanes for 161 cycles. The final read lengths for MiSeq and HiSeq were 300 nt and 160 nt in length. A total of 635,649,277 reads were assembled from the MiSeq/HiSeq pools. Quality control for reads generated from sequencing was performed using FastQC (http://www.bioinformatics.babraham.ac.uk/projects/fastqc/). Quality reads were used to perform de novo transcriptome assembly using Trinity. The initial assembly consisted of 187,216 genes. A filter was applied to keep only those genes that had at least 10 reads (across the 4 replicates) for an individual treatment. The resulting transcriptome contained 45,073 genes and reads. Functional annotation of the genes was done by using BLAST against *Arabidopsis thaliana*, *Zea mays*, and *Setaria italica*.

Differential gene expression analysis was performed using the R package, LIMMA (Ritchie et al. 2015). Within each time point, individual experimental treatments were compared to the control (ambient [CO_2_], ambient temperature). Following post ‘voom’ normalization, extreme outliers were removed, and 39,208 genes were assessed for differential expression analysis. Final normalized reads were log2 transformed for differential gene expression analysis. DESeq 2 (Love et al. 2014) was also run for comparison with the LIMMA approach and results were found to be similar but less conservative (Supplemental Fig. 1). A classical multidimensional scaling plot (MDS) was created using R base package with normalized and transformed reads. MDS plots were used to probe the relationships among samples in multidimensional space using a dissimilarity measure of each pairwise comparison.

### Metabolic profiling by GC–MS

Untargeted metabolite analysis was performed with pre-weighed lyophilized tissue (12.67–19.93 mg, average 16.93 ± 1.92 mg) according to protocol described in Ulanov and Widholm (Ulanov and Widholm 2010). For quality control (QC) 10 µl of leaf extract was taken from each sample and pooled, then run and analyzed after every 9 biological samples. Samples and QC were dried under vacuum and derivatized with 75 µl methoxyamine hydrochloride (Sigma-Aldrich, MO, USA) (40 mg ml^−1^ in pyridine) for 90 min at 50 °C, then with 125 μl MSTFA + 1%TMCS (Thermo, MA, USA) at 50 °C for 120 min followed by an additional 2-h incubation at room temperature. An internal standard (30 µL hentriacontanoic acid) was added to each sample prior to derivatization. Samples were analyzed on a gas chromatography/mass spectroscopy (GC/MS) system (Agilent Inc, Palo Alto, CA, USA) consisting of an Agilent 7890 gas chromatograph, an Agilent 5975 mass selective detector, and a HP 7683B autosampler. Gas chromatography was performed on a ZB-5MS capillary column (Phenomenex, Torrance, CA, USA). The inlet and MS interface temperatures were 250 °C, and the ion source temperature was adjusted to 230 °C. An aliquot of 1 µl was injected with the split ratio of 10:1. The helium carrier gas constant flow rate was 2.4 ml min^−1^. The temperature program was: 5 min isothermal heating at 70 °C, followed by an oven temperature increase of 50 °C min^−1^ to 310 °C, and a final 10 min at 310 °C. The mass spectrometer was operated in a positive electron impact mode at 69.9 eV ionization energy in m/z 50–800 scan range. The spectra of chromatogram peaks were compared with electron impact mass spectrum libraries NIST08 (NIST, MD, USA), WILEY08 (Palisade Corporation, NY, USA), and a custom library. To allow comparison among samples, data were normalized to the internal standard (hentriacontanoic acid, SIGMA, USA) and standardized to the dry weight of the individual sample. The chromatograms and mass spectra were evaluated using the AMDIS 2.71 (NIST, Gaithersburg, MD, USA) program, using a custom-built database. All known artificial peaks were identified and removed with prior data mining. The instrument variability was within the standard acceptance limit of 5%.

194 metabolites were identified with GC/MS. Technical replicates were averaged across samples. Normalized data was log10 transformed and univariate analysis was performed using SAS (SAS/STAT v9.4, SAS Institute, Inc.) to identify outliers. The MDS plot was created using R base package with normalized and transformed reads. The transformed and normalized metabolomics dataset was analyzed using a general linear model (ANOVA) with SAS PROC GLM where time point was determined non-significant (p > 0.01) and dropped from the model. Means separation tests were done using a two-tailed Dunnett, comparing each treatment to the control. Metabolites were considered significantly different from control if p < 0.01 (Table 1).

**Table 1:** List of metabolites with significant changes in the experimental treatments (p ≤ 0.01)

Compound	Ambient	Elevated [CO_2_]		Elevated Temperature		Combination	
	Log_2_(x̃) ± SE	Log_2_(x̃) ± SE	Log_2_(FC)	Log_2_(x̃) ± SE	Log_2_(FC)	Log_2_(x̃) ± SE	Log_2_(FC)
1,3-Diaminopropane	9.21 ± 0.27	9.81 ± 0.23	0.60	**10.91 ± 0.13**	***1.70***	9.95 ± 0.24	0.75
1-Benzylglucopyranoside	9.61 ± 0.21	**10.56 ± 0.17**	0.95	**10.89 ± 0.26**	***1.28***	**10.76 ± 0.15**	***1.15***
2-Methylmalic acid	11.27 ± 0.48	**8.25 ± 0.17**	*− 3.02*	9.60 ± 0.17	*− 1.67*	10.30 ± 0.38	*− 0.97*
α-ketoglutaric acid	9.93 ± 0.33	**7.78 ± 0.21**	*− 2.15*	10.57 ± 0.25	0.63	9.21 ± 0.25	*− *0.72
α-tocopherol	10.85 ± 0.23	11.44 ± 0.23	0.59	**12.25 ± 0.30**	*1.41*	**12.43 ± 0.16**	*1.58*
γ-tocopherol	8.48 ± 0.17	8.90 ± 0.14	0.42	**9.12 ± 0.18**	0.64	8.26 ± 0.12	*− *0.22
Arabinose	12.68 ± 0.12	13.21 ± 0.17	0.52	**13.92 ± 0.25**	***1.24***	13.34 ± 0.25	0.65
Arabitol	10.90 ± 0.22	11.61 ± 0.26*	0.71	**11.67 ± 0.14**	0.77	11.02 ± 0.16	0.12
Dehydroascorbic acid	11.54 ± 0.21	11.81 ± 0.14	0.27	**12.57 ± 0.15**	***1.02***	11.71 ± 0.19	0.17
Ethanolamine	14.58 ± 0.15	15.07 ± 0.13*	0.49	**15.23 ± 0.11**	0.65	14.70 ± 0.16	0.12
Fructose	20.19 ± 0.19	20.57 ± 0.21	0.38	**21.27 ± 0.11**	***1.08***	20.38 ± 0.23	0.19
Galactose	15.85 ± 0.18	**16.71 ± 0.21**	0.87	**16.88 ± 0.18**	***1.03***	16.22 ± 0.20	0.37
Gluconic acid	10.03 ± 0.05	10.68 ± 0.23	0.65	**10.98 ± 0.17**	0.95	10.77 ± 0.20	0.74
Glucose	20.55 ± 0.08	21.10 ± 0.21*	0.55	**21.47 ± 0.09**	0.92	20.54 ± 0.23	*− *0.01
Glyceric acid	16.47 ± 0.12	**17.20 ± 0.09**	0.72	**17.25 ± 0.15**	0.78	16.54 ± 0.20	0.07
Inositol	14.78 ± 0.18	15.50 ± 0.19	0.71	**15.83 ± 0.15**	***1.05***	15.47 ± 0.16	0.68
Isoleucine	9.59 ± 0.17	**10.40 ± 0.25**	0.81	**10.75 ± 0.22**	***1.16***	**10.58 ± 0.25**	***0.98***
Leucine	9.45 ± 0.33	10.11 ± 0.11	0.66	**10.95 ± 0.39**	***1.50***	**10.44 ± 0.35**	***0.98***
Maltose	15.06 ± 0.15	15.50 ± 0.14	0.44	**15.74 ± 0.07**	0.68	14.98 ± 0.26	*− *0.08
Melibiose	11.08 ± 0.23	11.91 ± 0.19	0.84	**13.02 ± 0.27**	***1.94***	**12.24 ± 0.37**	***1.17***
Neochlorogenic acid	16.72 ± 0.21	16.96 ± 0.11	0.24	**17.32 ± 0.14**	0.60	17.17 ± 0.13	0.45
O-acetylsalicylic acid	10.73 ± 0.11	11.00 ± 0.12	0.27	**11.79 ± 0.19**	***1.06***	**11.09 ± 0.22**	0.36
Oxalic acid	13.46 ± 0.16	13.54 ± 0.15	0.08	**11.92 ± 0.31**	*− 1.54*	**12.04 ± 0.31**	*− 1.42*
Phenylalanine	9.49 ± 0.13	9.83 ± 0.21	0.34	**10.76 ± 0.36**	***1.27***	**10.81 ± 0.40**	***1.31***
Quinic acid	17.30 ± 0.22	**18.22 ± 0.12**	0.92	**18.14 ± 0.12**	0.84	17.68 ± 0.13	0.39
Ribitiol	10.25 ± 0.25	10.81 ± 0.17	0.55	**11.57 ± 0.21**	***1.32***	10.90 ± 0.22*	0.64
Ribose	14.92 ± 0.17	15.25 ± 0.10	0.33	**15.56 ± 0.12**	0.64	15.13 ± 0.18	0.21
Serine	14.04 ± 0.11	13.92 ± 0.11	*− *0.12	**15.15 ± 0.16**	***1.11***	14.36 ± 0.26	0.32
Sinapic acid	7.30 ± 0.19	7.74 ± 0.15	0.45	**7.99 ± 0.20**	0.69	**7.98 ± 0.18**	0.69
Stigmasterol	11.52 ± 0.14	12.14 ± 0.25	0.62	**12.75 ± 0.22**	***1.23***	12.65 ± 0.15*	***1.13***
Threitol	8.97 ± 0.12	9.28 ± 0.09	0.31	**10.23 ± 0.15**	***1.26***	**9.78 ± 0.16**	0.81
Threonine	11.90 ± 0.29	12.38 ± 0.11	0.46	**13.30 ± 0.12**	***1.38***	12.77 ± 0.26	0.85
Valine	10.57 ± 0.40	11.19 ± 0.15*	0.61	**12.22 ± 0.18**	***1.64***	**11.91 ± 0.26**	***1.34***
Xylose	8.80 ± 0.20	9.71 ± 0.15*	0.91	**10.26 ± 0.26**	***1.46***	**10.08 ± 0.26**	***1.27***

The log_2_ normalized median values ± standard error and log_2_fold change (log_2_(FC)) for each treatment are shown. Metabolites significantly changed are denoted by bold value (*p* ≤ 0.01) or *(*p* ≤ 0.05). Bold italic cells show increased metabolite content and italics represents decreased metabolite content in the treatments

### Weighted gene correlations network analysis (WGCNA)

WGCNA was run separately for each time point (Langfelder and Horvath 2008). A signed network was performed with a power of 8 and 7 for time point A and B respectively. Metabolites were correlated to each module per time point, and physiological data was correlated for time point B. Time point B data did not perform well in the WGCNA network due to the variation within the data, this analysis was dropped. The genes present in each module were correlated with metabolites that were found significant in the ANOVA (p < 0.01), Supplemental File 1 shows the modules with DEG for time point A. The GOSlim-Plant was selected for the gene ontology classification with a Benjamini & Hochberg FDR correction of 0.01 annotated to *Arabidopsis thaliana.*

### Integration of metabolite and transcriptional data

Linear correlation analysis was performed in R, with the significantly differentially expressed transcripts and metabolites from the second time point (B), along with the physiological data collected and previously reported by Britto de Assis Prado et al. (2016). Linear correlation was selected to highlight possible relationships between the metabolite and transcript abundance. Additionally, the approach taken in time point A, using WGCNA, was not appropriate for the second time point because of high variation within replicates of a given treatment and because far fewer transcripts were significantly different among treatments (see results). Correlations with an adjusted *p* value (False Discovery rate) of 0.05 or less, and a correlation coefficient of 0.6 or greater were considered significant in this analysis.

## Results

### Transcriptome analysis

Sequencing produced a total of 635,649,777 paired-end reads, which were initially assembled into 187,216 contigs. Using a filter that kept only transcripts with at least 10 reads across the 4 replicates resulted in a final transcriptome containing 45,073 contigs with an average read length of 400 bp. Annotation of those contigs identified 27,502 unigenes with 11,538 BLASTX hits in the NCBI database. The transcriptome was classified into GO slim terms describing cellular components, molecular functions and biological processes (Supplemental Fig. 2). The highest abundance terms for cellular components, molecular functions, and biological processes were “cell”, “catalytic activity”, and “metabolic process” respectively, which was similar to another *Panicum maximum* transcriptome previously published (Toledo-Silva et al. 2013).

**Fig. 1:**
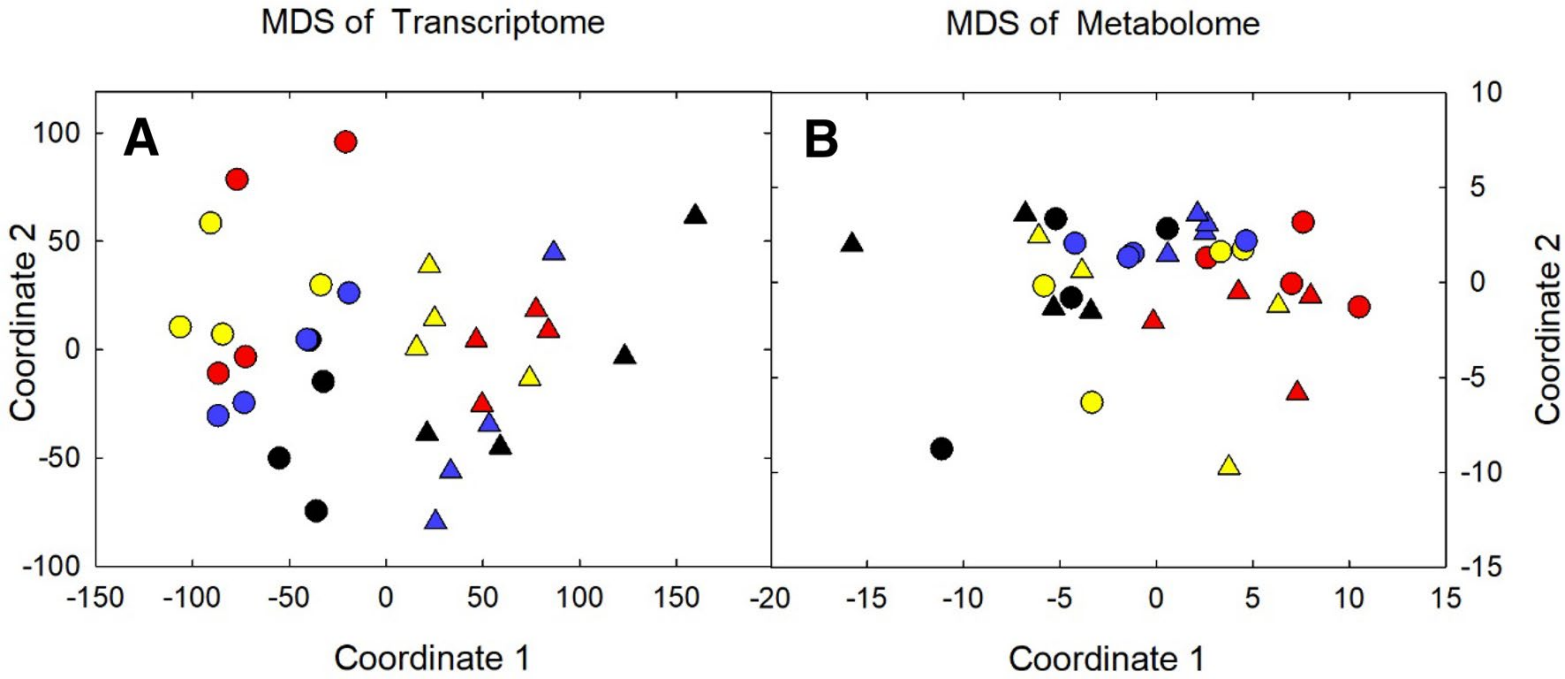
Multidimensional Scaling (MDS) plots for the transcript and metabolite experiments. **a** MDS plot using transcript data, showing a clear time point separation (circles vs triangles) as well as separation of elevated temperature and ambient temperature in Timepoint A (circles). **b** MDS plot using metabolite data, showing no clear separation of time points, but a clear separation of ambient treatment (black) from elevated temperature treatment (red). Time Point A (Circles), Time Point B (Triangles), Ambient (black), Elevated [CO_2_] (blue), Elevated Temperature (red), Combination (yellow)

Multidimensional scaling (MDS) analysis of leaf transcripts showed a clear time point separation and clustering of treatments within each time point (Fig. 1a). The treatment separation was less apparent in time point B and the variability within the control and eC plots was also greater (Fig. 1a). Differential transcript expression from time point A identified 666 transcripts that were altered in the eCeT treatment when compared to control (p < 0.1, Supplemental File 2). No transcripts were differentially expressed in the eC treatment, when compared to control, and only 3 transcripts were significantly altered in the eT treatment. GO slim terms were assigned to the 666 transcripts altered in the eCeT treatment. Overrepresentation tests were run to identify GO categories with increased occurrence within the differentially expressed genes, with a false discovery rate of 0.05. The most overrepresented GO categories were cellular component “ribosome”, molecular function “structural constituent of ribosome”, and biological process “protein folding” (Supplemental Fig. 3). Time point B showed the same trend as time point A, but with fewer transcripts significantly changing; only 71 total in eCeT, 2 in eT, and 0 in eC (p < 0.1, Supplemental File 2). The relaxed *p* value for DEG allowed a larger set of transcripts to test for correlation with metabolite content and/or leaf physiological traits. No GO categories were overrepresented within the significant transcripts in time point B.

### Metabolite analysis

Untargeted metabolomics was used to investigate biochemical changes in Guinea grass exposed to eC, eT and eCeT. Although 194 total metabolites were identified, only 125 metabolites were consistently present in the different treatments and those were used for analysis. MDS analysis revealed a separation of the eT treatment (red symbols) from the C plots (black symbols), but no clear separation between sampling dates (Fig. 1b). Additionally, the time points were not significantly different in the ANOVA (p > 0.01). A general linear model with a Dunnett’s test was run to test for significant differences in abundance among treatments, and 34 metabolites were significantly altered in at least one treatment compared to the C plots (p ≤ 0.01, Table 1). Only 1-benzylglucopyranoside and isoleucine were significantly different in each of the treatments compared to the C plots.

Overall the metabolome showed the greatest change in the eT treatment compared to C with 30 metabolites significantly changing (Table 1). All of the metabolites increased in abundance in eT relative to ambient, with the exception of 2-methylmalic acid and oxalic acid (Table 1). Melibiose showed the largest increase in eT amongst sugars, with a 1.94-fold change; arabinose, fructose, galactose, glucose maltose, ribose, and xylose were also significantly increased in eT. Notably sucrose was not altered under any treatment. Many amino acids showed significant increases under eT compared to ambient with Val Thr, and Phe increasing the most (1.64, 1.38, 1.27-fold; Table 1). Organic acids, including neochlorogenic acid and sinapic acid, which are involved in secondary metabolism and specifically monolignol metabolism showed significant increases under the eT treatment compared to C (Table 1).

The eC treatment did not affect the metabolome as much as eT, with only 2 metabolites showing a log2-fold ≥ |1| and p ≤ 0.01. 2-Methylmalic acid (citramalate) had a large decrease (− 3.02-fold) in the eC plots, and this compound decreased across all treatments. Additionally, α-ketoglutaric acid had a large decrease (− 2.15-fold) in eC. Ile was the only amino acid impacted in the eC plots, and was moderately increased by 0.81 fold (Table 1). Quinic acid, an important intermediate of the lignin pathway, increased in the eC plots (0.92-fold). 1-Benzylglucopyranoside had 0.95 log2-fold change in the eC plots.

The combination of eC and eT showed similar trends to the eT plots alone, but with a dampened effect (Table 1). All metabolites that had a significant change in the eCeT treatment were also significantly altered in the eT treatment. In both eT and eCeT, α-tocopherol increased significantly with a log2-fold > 1. Many compounds that were significantly increased in eT were not significantly altered in eCeT. For example, Ser and Thr were significantly increased in the eT treatment, but were not significantly impacted in eCeT, with a fold-change trend of decreasing in relative leaf content. Additionally, many sugars and sugar alcohols including; arabinose, fructose, galactose, glucose, and inositol increased in the eT plots but not in eCeT.

### Integration of metabolite and transcript data—timepoint A

Weighted gene correlation network analysis (WGCNA) was used to construct a signed network of clustered genes from the time point A (sampled after 30 days of treatment). Using 9802 transcripts, expression data were clustered into 9 unique modules. GO enrichment was performed for each module to identify the overrepresented biological functions within each grouping (Supplemental Table 1). Module 1 contained the largest number of transcripts (1719 total), but only 2 were differentially expressed in the eCeT treatments. Module 3 contained 948 transcripts, 84 of which were differently expressed in eCeT, and had significantly enrichment in regulation of salicylic acid biosynthesis process, defense to oomycetes, cell surface receptor signaling, plant-type hypersensitive response, protein phosphorylation, defense response to bacterium, and oxidation–reduction process. In addition, module 3 had a significant negative correlation with melibiose (Supplemental Table 1, Supplemental Fig. 4). Module 5 contained 484 transcripts, 35 of which were differently expressed in eCeT treatments, with significantly enrichment in oxylipin biosynthesis, terpenoid biosynthesis, response to wounding, hormone biosynthesis, secondary metabolite biosynthesis, defense response, and oxidation–reduction process.

**Fig. 2:**
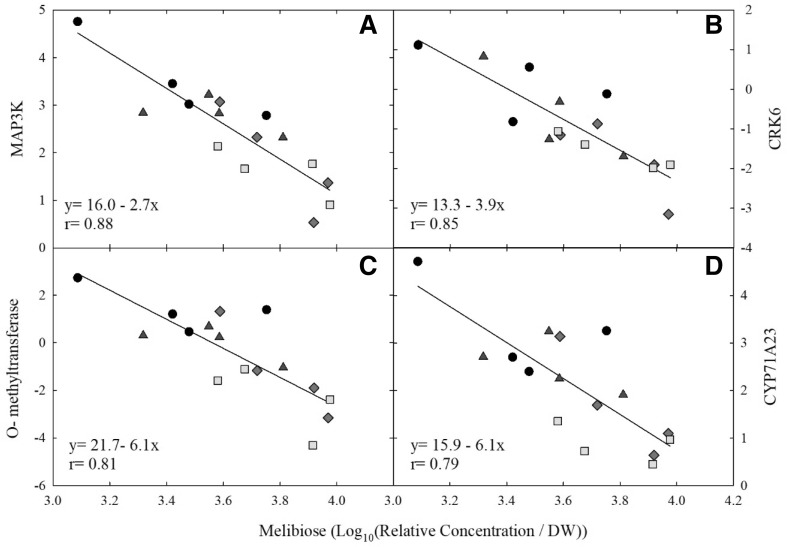
Linear correlation between gene expression and melibiose content. Significant linear relationships between melibiose and transcripts present within the weighted gene correlation network analysis module 3, for time point A (30 days of treatment). Correlations listed have |r| ≥ 0.70 and p < 0.01. Normalized expression values for each transcript are plotted on the y axis: **a** At1g54960–mitogen-activated protein kinase kinase kinase 2 (MAP3 K); **b** At4g23160–cysteine-rich receptor-like protein kinase 8 (CRK8); **c** At5g54160- *O*-methyltransferase; **d** At3g48300–cytochrome P450 (CYP71A23). Ambient (black, circle), elevated [CO_2_] (blue, triangle), elevated temperature (red, diamond), combination (yellow, square)

Within module 3, four transcripts were strongly correlated with melibiose content (r ≥ |0.8|, *p *< 0.01; Fig. 2). Melibiose showed significant negative correlations with the expression of two guard cell transcripts. The first, At4g23160, is annotated as cysteine-rich receptor-like protein kinase 8 (CRK8) and shows biological activity involved in defense response to bacterium and protein phosphorylation. The second was At3g48300, a cytochrome P450 (CYP71A23) involved in oxidation–reduction processes. The strongest relationship was identified for transcript ID, At1g54960, which was annotated as a subfamily of mitogen-activated protein kinase kinase kinase 2 (MAP3 K) known as Arabidopsis Nucleus- and Phragmoplast-Localized Kinase1 (NPK1)—related protein kinases (ANPs) specifically ANP2, in the *Arabidopsis* genome. The final gene with a negative linear relationship with melibiose was identified as transcript ID, At5g54160, a caffeic acid/5-hydroxyferulic acid O-methyltransferase 1 (COMT) with functions in lignin and flavonoid biosynthesis.

### Integration of metabolite and transcript data—timepoint B

**Fig. 3:**
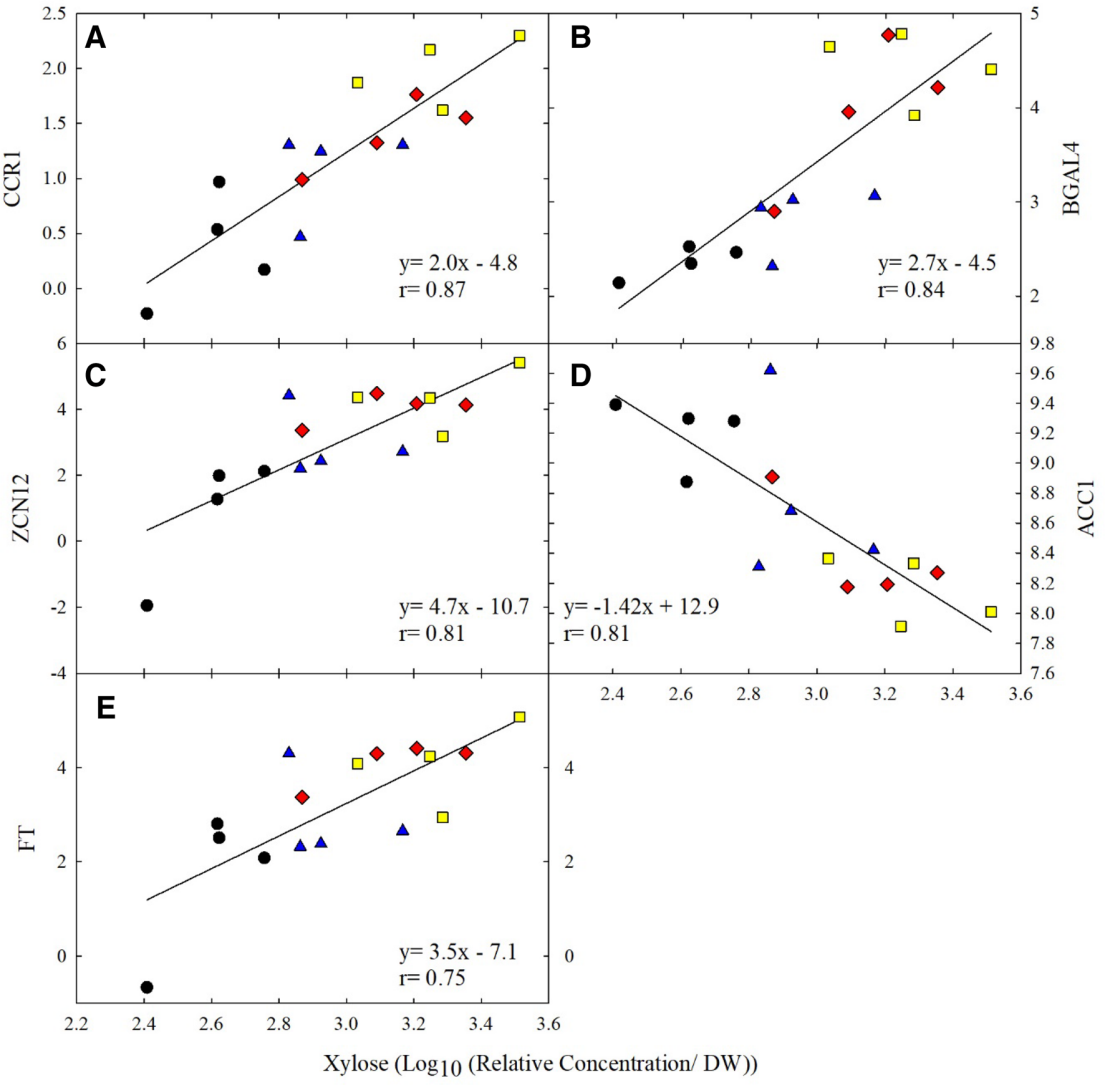
Linear correlation between gene expression and xylose content. Significant linear relationships between xylose content and transcript expression measured at time point B (50 days of treatment). Correlations listed have |r| ≥ 0.70 and p < 0.01. Normalized expression values for each transcript are plotted on the y axis: **a** At5g19440–NAD(P)-binding protein (CCR1); **b** At5g56870–β-galactosidase 4 (BGAL4); **c** At5g03840–terminal flower 1/Zea centroradialis (ZCN12); **d** At1g36160- acetyl-CoA carboxylase 1(ACC1); **e** At1g65480- flowering locus T (FT). Ambient (black, circle), elevated [CO_2_] (blue, triangle), elevated temperature (red, diamond), combination (yellow, square)

Several significant relationships were identified within the metabolite and transcript datasets, with a r ≥ |0.75| and an adjusted p ≤ 0.01 (Table 2). All significant linear correlations between DEG and metabolites are shown in Supplemental File 3. Xylose had the most significant linear correlations, 10 positive and one negative. The strongest relationship was between gene ID At5g19440 (r = 0.87) (Fig. 3). The BlastX annotation hit for *Arabidopsis thaliana* identified At5g19440 as a NAD(P)-binding protein, located in the cytosol, and involved in cinnamyl-CoA reductase (CCR) activity in a variety of biological processes including lignin biosynthesis and response to cold. This sequence was highly similar to the annotated CCR1 in the *Z. mays* genome (LOC100382657) and predicated CCR1 in *S.italica* genome (LOC101786254).

**Table 2:** Number of significant linear correlations between metabolite content and gene expression measured at time point B (50 d of treatment)

Metabolite	Positive	Negative
1-Benzylglucopyranoside	1	0
α-Tocopherol	2	0
β-Sitosterol	1	0
*O*-acetylsalicylic acid	2	0
Sinapic acid	4	1
Stigmasterol	1	0
Valine	2	0
Xylose	10	1

Correlations have |r| ≥ 0.75 and *p *< 0.01

The second strongest relationship between a transcript and xylose was transcript At5g56870 (r = 0.84). This transcript was annotated as β-galactosidase 4 (BGAL4) in the *Arabidopsis* genome. This gene is localized to the cytoplasm and functions in rRNA N-glycosylase activity and defense response, also acting as a negative regulator of translation. Along with the strong linear correlation to xylose, BGAL4 was also significantly correlated with α-tocopherol, stigmasterol, and O-acetylsalicylic acid (r = 0.80, 0.76, 0.75, respectively; Supplemental File 3). Two additional transcripts, At5g03840 (r = 0.81) and At1g36160 (r = − 0.81), showed significant linear correlations with xylose content. Transcript ID At5g03840 was annotated as terminal flower 1 (TFL1) controlling inflorescence meristem identity within the *Arabidopsis* genome and showed 73% homolog with the *Zea mays* ZCN12 (*Zea* centroradialis (ZCN)) (NM_001112779.2). This gene functions in many biological roles including acting as a negative regulator of cell aging and is expressed during growth and development stages from flowering to leaf senescence. ZCN12 was also found to have strong correlations with sinapic acid and valine (Supplemental File 3). Unlike the first three gene correlations, At1g36160, showed a negative linear correlation with xylose and sinapic acid (r = − 0.74). This gene was annotated in the Arabidopsis genome as acetyl-CoA carboxylase 1 (ACC1), which regulates the initial step in the fatty acid biosynthesis pathway of acetyl-CoA to malonyl-CoA.

In addition to the strong associations with xylose (Fig. 3e), transcript ID At1g65480 was correlated with 4 additional metabolites. The BlastX annotation hit for *Arabidopsis thaliana* identified this transcript as flowering locus T (FLT) functioning in phosphatidylethanolamine binding with biological processes in flower development. The Blast annotation for *Zea mays* had only 52% homology with TWIN SISTER of FT (NM_001309850.1) and predicted *Setaria italica* FLT (XM_004960683.3) with a homology of 53%. Positive linear correlations were identified between FLT and valine, sinapic acid, O-acetylsalicylic acid, β- sitosterol, and xylose (r = 0.84, 0.82, 0.78, 0.77, 0.75) respectively (Supplemental Fig. 5).

## Discussion

This study investigated the transcriptional and metabolite response of Guinea grass to growth at eC, eT and the combination of eCeT under open-air field conditions. Previous physiological analysis of the plants grown at this FACE experiment revealed that growth at eT increased leaf biomass and leaf area, but there was considerable variation within each treatment (Supplemental Fig. 6) (Britto de Assis Prado et al. 2016). The transcript and metabolite data also showed significant variation within each treatment, revealed by MDS (Fig. 1), but different groupings were apparent in the transcript and metabolite data. The transcriptome showed a clear separation among sampling dates (30 or 50 days of treatment), while the metabolome data showed a clear separation of treatments, most clearly C and eT (Fig. 1). The transcriptome also showed separation of treatments at the first sampling date (Timepoint A), but not the second (Timepoint B) (Fig. 1a). The within-treatment variability observed in the data may be due to environmental and edaphic properties of the field site (i.e., soil characteristics and/or slope), which could not be accounted for in the analysis, or from the relatively low sample size (n = 4). However, the fact that no transcripts were significantly altered in response to eC alone is consistent with previous experiments done with other C_4_ grasses, which showed that in the absence of drought stress, there is no direct effect of elevated [CO_2_] on C_4_ metabolism (Wall et al. 2001; Leakey et al. 2006). On the other hand, eT stimulated the growth of *P.maximum* in this study (Supplemental Fig. 6), yet only a few genes were significantly differentially expressed in eT alone. Optimal growth for *P. maximum* is estimated to be at least 21 °C, and background temperature in ambient conditions during this experiment was only 17.0 ± 0.06 °C, so the eT treatment moved the plants closer to optimal growth temperatures (Araujo et al. 2013). In the combination (eCeT) treatment, significant changes in the transcriptome were observed, most notably transcripts involved in protein translation and assembly.

A key aim of this work was to integrate the transcript and metabolite data sets. Because of the clear differences in the transcript data between time points A and B (Fig. 1a), integration was done within each time point and not across time points. In addition, time point B offered the potential to integrate leaf physiological data with the transcript and metabolite datasets. Because of the large level of variability within individual treatments for all of the parameters, linear correlation analysis was used for data integration. For time point A WGCNA networks were built to group genes responding in similar ways together to form a larger network. These networks were then correlated with the metabolite data to identify interactions of interest. Because there were fewer DEG within time point B, WGCNA was not possible, and so for time point B, simple linear correlations between the DEG, significant metabolites, and leaf physiological data were used to identify correlations of interest.

### Integration of metabolomic and transcript data—time point A

Two modules (3 and 5) from the WGCNA analysis contained significant numbers of DEG (Supplemental Table 2). The DEG identified within module 3 were enriched in salicylic acid (SA) biosynthesis (*p* 0.016) and general defense response to bacteria, suggesting greater stress in the eCeT treatment compared to control. The enrichment of SA biosynthesis transcripts has previously been identified to be part of the heat-stress response in plants. Salicylic acid stabilizes heat shock transcription factors increasing their binding to heat shock elements and thus promoting heat shock-related genes (Wahid et al. 2007). While the background temperature during the experiments was low, it is possible that the combination of eC and eT increased leaf temperatures more than just the eT temperature alone, thus triggering some stress-related transcriptional responses. The GO-term enrichment analysis of the transcripts associated with module 5 are also in agreement with a trend towards up-regulation of secondary metabolites involved in defense response.

Many amino acids are precursors to secondary metabolites that change in response to various environmental perturbations. In this study, the eT and eCeT treatments resulted in significantly greater levels of amino acids derived from oxaloacetate and pyruvate. Accumulation of such amino acids is well known in abiotic stresses and heat (Joshi et al. 2010; Kaplan et al. 2004; Rizhsky et al. 2004; Guy et al. 2008; Obata and Fernie 2012; Obata et al. 2015). The role of branched chain amino acids and other amino acids in improving heat tolerance is complex and not fully understood, but likely provides support for an increase in production of secondary metabolites to influence defense response during temperature stress (Kaplan et al. 2004; Du et al. 2011). Additionally, increases in amino acid pools have the potential to serve as osmolytes under heat stress (Joshi et al. 2010) or an alternative electron donor for the mitochondrial electron transport chain (Araujo et al. 2013). High levels of Ile, Val, and Phe were observed in the leaves of Bermuda grass and mature kernels of rice exposed to elevated temperature and were thought to improve heat tolerance (Du et al. 2011; Yamakawa and Hakata 2010; Kaplan et al. 2004). The buildup of transcripts in the brown and green modules involved in defense and secondary metabolite biosynthesis in combination with the increase in amino acids suggest up-regulation of defense metabolism in the eCeT treatment at 30 days post-treatment exposure.

Plant grown under abiotic stress condition use compatible osmolytes, low molecular mass compounds, to provide an adaptive mechanism. Accumulation of these soluble sugars such as fructose, sucrose, and glucose are documented to signal stress conditions and improve tolerance to cold, heat, and drought stress ((Du et al. 2011; Wahid et al. 2007; Kaplan et al. 2004; Rizhsky et al. 2004). The eT plots, regardless of [CO_2_], showed increased soluble sugar content; including melibiose. Melibiose is a common osmolyte and a byproduct of the hydrolysis of raffinose, via invertase (ElSayed et al. 2014), and has been identified to accumulate under various environmental conditions including drought stress and elevated temperature in both C_3_ and C_4_ species (Rizhsky et al. 2004; Kaplan et al. 2004; Du et al. 2011). Figure 2 shows the relationship of four transcripts negatively associated with melibiose, the strongest of which was MAP3 K. MAPKs are highly conserved signaling modules in eukaryotes involved in cellular responses, including response to stimuli. These cascades trigger activation of transcription factors, via phosphorylation, and other cellular responses to signal a stimulus response (Xu and Zhang 2015). This transcript was annotated as ANP2/NPK1; these enzymes activate stress-related MAPKs activated by ROS (Kovtun et al., 2000). In this study, there was an inverse relationship between MAP3 K expression and melibiose content, suggesting that there was less stress-related signaling in the eT plots.

The second transcript highly associated with melibiose was a caffeic acid/5-hydroxyferulic acid O-methyltransferase 1 (COMT). Switchgrass and maize mutants have shown that downregulation of COMT impacts lignin content of biofuel crops specifically decreasing the S-lignin units and improving ethanol yields (Liu et al. 2017; Guillaumie et al. 2008). Downregulation of this transcript could signal a shift from lignin biosynthesis to secondary defense metabolism.

The final two transcripts with a strong negative relationship to melibiose were CRK6 and CYP71A23, annotated as CYP72A26 in the maize genome (Jameson et al. 2008). Both of these transcripts are expressed in the guard cells. CRKs are induced by pathogen infection, and transgenic *Arabidopsis* plants with significantly elevated levels of CRK5 had enhanced leaf growth and increased pathogen resistance (Chen et al. 2003). CRK6 was found to be involved in ROS mediated signal processes with increased transcript abundance after exposure to pathogens, SA, and ROS (Chen et al. 2003, 2004). The decrease in abundance of transcripts involved in guard cell metabolism is interesting and could potentially indicate a stomatal response within the eT and eTeC plots. The correlation between transcripts that are tightly regulated with complex feedback interactions between environment and cellular metabolism and melibiose lead us to suggest that melibiose functions in osmotic adjustment under eT and eTeC to protect cellular components and enhance acclimation to eT.

### Integration of growth, metabolomic, and transcript data—timepoint B

Time Point B (50 d of treatment) allowed us to test the correlation of leaf biomass and area with metabolite and transcript levels; however, no significant correlations were observed. The low temperatures reported on the day of sampling may have greatly affected the metabolite and transcript levels, hindering the correlation of these datasets with leaf measurements taken at the end of the season. Field conditions are also inherently more variable than controlled environment conditions, but field experiments better represent the variation expected in the future. After 50 d of treatment, we did observe significant correlations between transcripts and metabolites, with the most notable being xylose (Table 2). Xylose is a monosaccharide and a precursor to pyruvate and acetyl-CoA.

The use of linear correlation analysis allowed for the identification of associations that might not have been predicted with traditional methods. Three transcripts, shown in Fig. 3, had a strong positive correlation with xylose content. The strongest correlation was with *Z. mays* cinnamoyl-CoA reductase (CCR1). CCR1 is the entry point into the lignin branch of the phenylpropanoid pathway (Piquemal et al. 1998). The position of CCR in the phenylpropanoid pathway gives this enzyme direct control over the direction of the metabolic flux toward flavonoids or monolignols(Sattler et al. 2017). *ZmCCR1* mutants with a moderate down-regulation of CCR1 activity increased the digestibility of cell walls without severely modifying lignin content (Tamasloukht et al. 2011). The overexpression of *SbMyb60* transcription factor in *Sorghum bicolor* was found to directly impact the monolignol biosynthesis with higher abundance of syringyl lignin, cell wall composition, with increases in soluble phenolic and aromatic amino acids (Scully et al. 2016, 2018). The observed increase in abundance of sinapic acid, phenylalanine, and α-Tocopherol under the eT treatment, regardless of [CO_2_], point to an impact of eT on lignin composition, possibly increased S-type concentrations, although that would need to be tested. In addition to components of cell wall structure, monolignol polymers have been induced under various biotic and abiotic stress conditions (Tronchet et al. 2010; Vincent et al. 2005). A similar heating experiment with both C_4_ and C_3_ grasses was performed at the prairie heating and CO_2_ enrichment site located in Wyoming, USA. A metabolomic profiling of *Bouteloua gracilis*, a C_4_ grass, identified similar responses including an increase in phenolic and sugar content within the senesced leaves of plants grown under elevated temperature and the combination treatment, along with an increase in amino acid content under the elevated temperature with ambient [CO_2_] (Suseela et al. 2014).

Sugars are continuously moving thru the phloem from source to sink and convey signaling information along the way, including regulation of flowering time (Gibson 2005). In several species adding exogenous sucrose and possibly other sugars promotes flowering (Cho et al. 2018). Interestingly, xylose was identified to have two significant positive correlations with flowering time-related genes, FT and ZCN12 (AtTFL1, Fig. 3c, d). The *Arabidopsis* orthologs are two closely related genes that are key integrators of flowering transition pathways. These two genes have antagonistic functions in the flower transition pathway; FT promotes flowering and TFL1 suppresses the transition (Shannon and Meekswagner 1991). Unlike the ortholog gene, ZCN12 belongs to the FT-like II group, primarily expressed in the leaf blades, and is activated after floral transition and is continually express thru late stages of vegetative development (Danilevskaya et al. 2010). FT-like proteins mediate numerous processes including growth, plant architecture, fruiting and tuber formation (Pin and Nilsson 2012). Recent evidence has shown FT can act as a cell autonomous regulator of stomatal guard cell opening and closure (Kinoshita et al. 2011). Kinoshita and colleagues showed overexpression of FT in *Arabidopsis* increased H^+^-ATPases activity within the guard cells, suggesting that FT likely influences the stomatal opening/closure via regulation of H^+^-ATPase. The accumulation of FT related transcripts within eT and eCeT could offer an additional regulation of stomatal function correlating with the osmotic adjustment provided from the accumulation of soluble sugars, such as melibiose and xylose.

## Conclusion

This study investigated tropical Guinea grass response to global atmospheric change and rising temperature in the field. Although warming treatments in temperate regions often decreased productivity, heating the canopy 1.5 °C above ambient in this experiment resulted in increased leaf area and biomass. Field transcriptomics and metabolomics identified metabolic pathways that were altered by growth at elevated [CO_2_] and temperature. Melibiose and xylose content were greater in plants exposed to elevated temperature, and content of these sugars was significantly correlated to the expression of genes involved in secondary metabolism, defense response, and stomatal function. We hypothesize that the metabolite and transcript responses were associated with alleviation of stress and greater growth at elevated temperature.

## Electronic supplementary material

Below is the link to the electronic supplementary material.

Supplementary material 1 (CSV 85 kb)

Supplementary material 2 (CSV 57 kb)

Supplementary material 3 (CSV 8 kb)

Supplementary material 4 (DOCX 967 kb)

## Data Availability

The metabolomics and metadata reported in this paper are available via the NIH Common Fund’s Data Repository and Coordinating Center (supported by NIH grant, U01-DK097430) website, the Metabolomics Workbench, http://www.metabolomicsworkbench.org, where it has been assigned Project ID (PR000717). The data can be accessed directly via it’s Project DOI: 10.21228/M80M50. The transcriptomic and metadata reported in this publication have been deposited in NCBI’s Gene Expression Omnibus and are accessible through GEO series accession number GSE122194 (https://www.ncbi.nlm.nih.gov/geo/query/acc.cgi?acc=GSE122194https://www.ncbi.nlm.nih.gov/geo/query/acc.cgi?acc=GSE122194).

## References

[CR1] Araujo LC, Santos PM, Rodriguez D, Pezzopane JRM, Oliveira PPA, Cruz PG (2013). Simulating guinea grass production: Empirical and mechanistic approaches. Agronomy Journal.

[CR2] Bilgin DD, DeLucia EH, Clough SJ (2009). A robust plant RNA isolation method suitable for Affymetrix GeneChip analysis and quantitative real-time RT-PCR. Nature Protocols.

[CR3] Boval M, Dixon RM (2012). The importance of grasslands for animal production and other functions: A review on management and methodological progress in the tropics. Animal.

[CR100] Cai C, Yin X, He S, Jiang W, Si C, Struik PC (2016). Responses of wheat and rice to factorial combinations of ambient and elevated CO_2_ and temperature in FACE experiments. Global Change Biology.

[CR101] Cardoso AS, Berndt A, Leytem A, Alves BJR, de Carvalho IDO, Soares LHD (2016). Impact of the intensification of beef production in Brazil on greenhouse gas emissions and land use. Agricultural Systems.

[CR4] Chen KG, Du LQ, Chen ZX (2003). Sensitization of defense responses and activation of programmed cell death by a pathogen-induced receptor-like protein kinase in Arabidopsis. Plant Molecular Biology.

[CR5] Chen KG, Fan BF, Du LQ, Chen ZX (2004). Activation of hypersensitive cell death by pathogen-induced receptor-like protein kinases from Arabidopsis. Plant Molecular Biology.

[CR102] Cho LH, Pasriga R, Yoon J, Jeon JS, An G (2018). Roles of sugars in controlling flowering time. Journal of Plant Biology.

[CR6] Danilevskaya ON, Meng X, Ananiev EV (2010). Concerted modification of flowering time and inflorescence architecture by ectopic expression of TFL1-like genes in maize. Plant Physiology.

[CR7] de Assis Britto, Prado CH, Guedes de Camargo-Bortolin L, Castro E, Martinez CA (2016). Leaf dynamics of Panicum maximum under future climatic changes. PLoS ONE.

[CR104] Dieleman WIJ, Vicca S, Dijkstra FA, Hagedorn F, Hovenden MJ, Larsen KS (2012). Simple additive effects are rare: A quantitative review of plant biomass and soil process responses to combined manipulations of CO_2_ and temperature. Global Change Biology.

[CR8] Du HM, Wang ZL, Yu WJ, Liu YM, Huang BR (2011). Differential metabolic responses of perennial grass *Cynodon transvaalensis* x *Cynodon dactylon* (C4) and *Poa pratensis* (C3) to heat stress. Physiologia Plantarum.

[CR9] ElSayed AI, Rafudeen MS, Golldack D (2014). Physiological aspects of raffinose family oligosaccharides in plants: Protection against abiotic stress. Plant Biology.

[CR10] Gibson SI (2005). Control of plant development and gene expression by sugar signaling. Current Opinion in Plant Biology.

[CR11] Guillaumie S, Goffner D, Barbier O, Martinant JP, Pichon M, Barriere Y (2008). Expression of cell wall related genes in basal and ear internodes of silking brown-midrib-3, caffeic acid O-methyltransferase (COMT) down-regulated, and normal maize plants. BMC Plant Biology.

[CR12] Guy C, Kaplan F, Kopka J, Selbig J, Hincha DK (2008). Metabolomics of temperature stress. Physiologia Plantarum.

[CR13] Jameson N, Georgelis N, Fouladbash E, Martens S, Hannah LC, Lal S (2008). Helitron mediated amplification of cytochrome P450 monooxygenase gene in maize. Plant Molecular Biology.

[CR14] Joshi V, Joung JG, Fei ZJ, Jander G (2010). Interdependence of threonine, methionine and isoleucine metabolism in plants: Accumulation and transcriptional regulation under abiotic stress. Amino Acids.

[CR15] Kaplan F, Kopka J, Haskell DW, Zhao W, Schiller KC, Gatzke N (2004). Exploring the temperature-stress metabolome of Arabidopsis. Plant Physiology.

[CR16] Kinoshita T, Ono N, Hayashi Y, Morimoto S, Nakamura S, Soda M (2011). FLOWERING LOCUS T regulates stomatal opening. Current Biology.

[CR17] Kovtun Y, Chiu WL, Tena G, Sheen J (2000). Functional analysis of oxidative stress-activated mitogen-activated protein kinase cascade in plants. Proceedings of the National academy of Sciences of the United States of America.

[CR18] Langfelder P, Horvath S (2008). WGCNA: an R package for weighted correlation network analysis. BMC Bioinformatics.

[CR105] Langridge P, Fleury D (2011). Making the most of ‘omics’ for crop breeding. Trends in Biotechnology.

[CR106] Leakey ADB, Bishop KA, Ainsworth EA (2012). A multi-biome gap in understanding of crop and ecosystem responses to elevated CO_2_. Current Opinion in Plant Biology.

[CR19] Leakey ADB, Uribelarrea M, Ainsworth EA, Naidu SL, Rogers A, Ort DR (2006). Photosynthesis, productivity, and yield of maize are not affected by open-air elevation of CO_2_ concentration in the absence of drought. Plant Physiology.

[CR20] Liu SJ, Fu CX, Gou JQ, Sun L, Huhman D, Zhang YW (2017). Simultaneous downregulation of MTHFR and COMT in switchgrass affects plant performance and induces lesion-mimic cell eeath. Frontiers in Plant Science.

[CR21] Love MI, Huber W, Anders S (2014). Moderated estimation of fold change and dispersion for RNA-seq data with DESeq2. Genome Biology.

[CR107] Lovell JT, Shakirov EV, Schwartz S, Lowry DB, Aspinwall MJ, Taylor SH (2016). Promises and challenges of eco-physiological genomics in the field: Tests of drought responses in Switchgrass. Plant Physiology.

[CR108] Meyer E, Aspinwall MJ, Lowry DB, Palacio-Mejia JD, Logan TL, Fay PA (2014). Integrating transcriptional, metabolomic, and physiological responses to drought stress and recovery in switchgrass (*Panicum virgatum L.*). BMC Genomics.

[CR22] Obata T, Fernie AR (2012). The use of metabolomics to dissect plant responses to abiotic stresses. Cellular and Molecular Life Sciences.

[CR23] Obata T, Witt S, Lisec J, Palacios-Rojas N, Florez-Sarasa I, Yousfi S (2015). Metabolite profiles of maize leaves in drought, heat, and combined stress field trials reveal the relationship between metabolism and grain yield. Plant Physiology.

[CR109] Pedreira BC, Pedreira CGS, Lara MAS (2015). Leaf age, leaf blade portion and light intensity as determinants of leaf photosynthesis in *Panicum maximum* Jacq. Grassland Science.

[CR110] Piquemal J, Lapierre C, Myton K, O’Connell A, Schuch W, Grima-Pettenati J (1998). Down-regulation of cinnamoyl-CoA reductase induces significant changes of lignin profiles in transgenic tobacco plants. Plant Journal.

[CR24] Pin PA, Nilsson O (2012). The multifaceted roles of FLOWERING LOCUS T in plant development. Plant, Cell and Environment.

[CR25] Ramankutty N, Evan AT, Monfreda C, Foley JA (2008). Farming the planet: 1. Geographic distribution of global agricultural lands in the year 2000. Global Biogeochemical Cycles.

[CR26] Ritchie ME, Phipson B, Wu D, Hu YF, Law CW, Shi W (2015). limma powers differential expression analyses for RNA-sequencing and microarray studies. Nucleic Acids Research.

[CR27] Rizhsky L, Liang HJ, Shuman J, Shulaev V, Davletova S, Mittler R (2004). When defense pathways collide. The response of Arabidopsis to a combination of drought and heat stress. Plant Physiology.

[CR111] Ruiz-Vera UM, Siebers MH, Drag DW, Ort DR, Bernacchi CJ (2015). Canopy warming caused photosynthetic acclimation and reduced seed yield in maize grown at ambient and elevated CO_2_. Global Change Biology.

[CR112] Ryan EM, Ogle K, Zelikova TJ, LeCain DR, Williams DG, Morgan JA (2015). Antecedent moisture and temperature conditions modulate the response of ecosystem respiration to elevated CO_2_ and warming. Global Change Biology.

[CR28] Sattler SA, Walker AM, Vermerris W, Sattler SE, Kang C (2017). Structural and biochemical characterization of cinnamoyl-CoA reductases. Plant Physiology.

[CR29] Scully ED, Gries T, Palmer NA, Sarath G, Funnell-Harris DL, Baird L (2018). Overexpression of SbMyb60 in *Sorghum bicolor* impacts both primary and secondary metabolism. New Phytologist.

[CR30] Scully ED, Gries T, Sarath G, Palmer NA, Baird L, Serapiglia MJ (2016). Overexpression of SbMyb60 impacts phenylpropanoid biosynthesis and alters secondary cell wall composition in Sorghum bicolor. Plant Journal.

[CR31] Shannon S, Meekswagner DR (1991). A mutation in the Arabidopsis TFL1 gene affects inflorescence meristem development. Plant Cell.

[CR114] Sicher RC, Barnaby JY (2012). Impact of carbon dioxide enrichment on the responses of maize leaf transcripts and metabolites to water stress. Physiologia Plantarum.

[CR32] Still CJ, Berry JA, Collatz GJ, DeFries RS (2003). Global distribution of C-3 and C-4 vegetation: Carbon cycle implications. Global Biogeochemical Cycles.

[CR115] Sui N, Yang Z, Liu ML, Wang BS (2015). Identification and transcriptomic profiling of genes involved in increasing sugar content during salt stress in sweet sorghum leaves. BMC Genomics.

[CR33] Sun JF, Xia ZW, He TX, Dai WW, Peng B, Liu J (2017). Ten years of elevated CO_2_ affects soil greenhouse gas fluxes in an open top chamber experiment. Plant and Soil.

[CR34] Suseela V, Triebwasser-Freese D, Linscheid N, Morgan JA, Tharayil N (2014). Litters of photosynthetically divergent grasses exhibit differential metabolic responses to warming and elevated CO_2_. Ecosphere.

[CR35] Svensson BM, Carlsson BA, Melillo JM (2018). Changes in species abundance after seven years of elevated atmospheric CO_2_ and warming in a Subarctic birch forest understorey, as modified by rodent and moth outbreaks. Peer J.

[CR36] Tamasloukht B, Lam M, Martinez Y, Tozo K, Barbier O, Jourda C (2011). Characterization of a cinnamoyl-CoA reductase 1 (CCR1) mutant in maize: Effects on lignification, fibre development, and global gene expression. Journal of Experimental Botany.

[CR37] Toledo-Silva G, Cardoso-Silva CB, Jank L, Souza AP (2013). De novo transcriptome assembly for the tropical grass *Panicum maximum* Jacq. PLoS ONE.

[CR38] Tronchet M, Balague C, Kroj T, Jouanin L, Roby D (2010). Cinnamyl alcohol dehydrogenases-C and D, key enzymes in lignin biosynthesis, play an essential role in disease resistance in Arabidopsis. Molecular Plant Pathology.

[CR39] Ulanov A, Widholm JM (2010). Metabolic profiling to determine the cause of the increased triphenyltetrazolium chloride reduction in mannitol-treated maize callus. Journal of Plant Physiology.

[CR40] Vincent D, Lapierre C, Pollet B, Cornic G, Negroni L, Zivy M (2005). Water deficits affect caffeate O-methyltransferase, lignification, and related enzymes in maize leaves. A proteomic investigation. Plant Physiology.

[CR41] Wahid A, Gelani S, Ashraf M, Foolad MR (2007). Heat tolerance in plants: An overview. Environmental and Experimental Botany.

[CR42] Wall GW, Brooks TJ, Adam R, Cousins AB, Kimball BA, Pinter PJ (2001). Elevated atmospheric CO_2_ improved Sorghum plant water status by ameliorating the adverse effects of drought. New Phytologist.

[CR43] Xu J, Zhang SQ (2015). Mitogen-activated protein kinase cascades in signaling plant growth and development. Trends in Plant Science.

[CR44] Yamakawa H, Hakata M (2010). Atlas of rice grain filling-related metabolism under high temperature: Joint analysis of metabolome and transcriptome demonstrated inhibition of starch accumulation and induction of amino acid accumulation. Plant and Cell Physiology.

