# Supplementary material for: Metabolite and transcript profiling of Guinea grass (Panicum maximum Jacq) response to elevated [CO2] and temperature
Source: Metabolomics. 2019 Mar 25;15(4):51. doi: 10.1007/s11306-019-1511-8 (PMC6434026; doi:10.1007/s11306-019-1511-8)
Supplement: Supplementary file 4 — Supplementary material 4 (DOCX 967 kb) [file 11306_2019_1511_MOESM4_ESM.docx]

**Supplemental Content**

Metabolite and transcript profiling of Guinea grass (*Panicum maximum* Jacq) response to elevated [CO_2_] and temperature

*Metabolomics*

Jessica M. Wedow, Craig R. Yendrek, Tathyana R. Mello, Silvana Creste, Carlos A. Martinez, Elizabeth A. Ainsworth^1,2*^

^1^ Department of Plant Biology & Carl R. Woese Institute for Genomic Biology, University of Illinois at Urbana-Champaign

^2^ USDA Agricultural Research Service, Global Change and Photosynthesis Research Unit, Urbana, IL

^*^Corresponding Author: Elizabeth A. Ainsworth, 1201 W. Gregory Drive, 147 ERML, Urbana, IL 61801 USA, lisa.ainsworth@ars.usda.gov

**Supplemental File 1.** Differentially expressed genes (DEG) within each weighted gene correlations network analysis (WGCNA) module for time point A. Values show the elevated CO_2_ and elevated temperature (eCeT) treatment compared to ambient control (C) based on LIMMA analysis.

**Supplemental File 2:** Differentially expressed genes (DEGs) in combined elevated CO_2_ and elevated temperature (eCeT) treatments from time points A and B. The log2 fold change (log2FC) is shown along with statistical analysis of differential gene expression.

**Supplemental File 3:** Linear correlation coefficient (r) and significance (Adj.P) of linear relationships between metabolites and transcripts sampled at Time Point B.

**Supplemental Table 1: Weighted gene correlation network analysis (WGCNA) signed network statistics for time point A with GO-term enrichment analysis.** Minimum module size was 75 genes. The genes contained within a module were used to identify significantly increased GO-Enrichment biological process terms (*P*<0.01). Metabolites listed were significantly correlated with the corresponding module (*P*<0.05, |r| ≥ 0.75). *Module: WGCNA gene grouping, Number of Genes: total number of genes within each module, Number of DEG: total number of differentially expressed genes within each module, Metabolite (P,r): Linear correlation between module eigengen based connectivity and gene significance for metabolite (p value and correlation coefficient), GO-term enrichment: gene ontology enrichment of total number of genes within the module.*

| **Module** | **Number**  **of Genes** | **Number**  **of DEG** | **Metabolite**  **(*P*, r)** | **GO-term Enrichment**  **(GO-term ID; Fold Enrichment)** |
| --- | --- | --- | --- | --- |
| 1 | 1719 | 2 | - | Phosphate-containing compound metabolic process  (GO:0006796; 1.67) |
| 2 | 1260 | 9 | - | Gibberellin biosynthetic process  (GO:0009686; 12.82)  Cell surface receptor signaling  (GO:0007166; 3.81)  Protein phosphorylation  (GO:0006468; 2.74) |
| 3 | 948 | 84 | Melibiose  (5.8e-04, -0.76) | Defense response to oomycetes  (GO:0002229; 12.7)  Transmembrane receptor protein tyrosine kinase signaling  (GO:0007169; 6.94)  Response to bacterium  (GO:0009617; 6.94) |
| 4 | 536 | 2 | - | Protein phosphorylation  (GO:0006468; 2.71)  Defense response  (GO:0006952; 2.19) |
| 5 | 484 | 35 | - | Oxylipin biosynthesis  (GO:0031408; 17.06)  Terpenoid biosynthesis  (GO:0046246; 9.22)  Response to wounding  (GO:0009611; 5.03)  Hormone biosynthesis  (GO:0042446; 4.24)  Secondary metabolite biosynthesis  (GO:0044550; 3.85)  Defense response to other organisms  (GO:0098542; 2.28)  Oxidation-reduction process  (GO:0055114; 2.23) |
| 6 | 444 | 2 | - | Response to wounding  (GO:0009611; 6.20) |


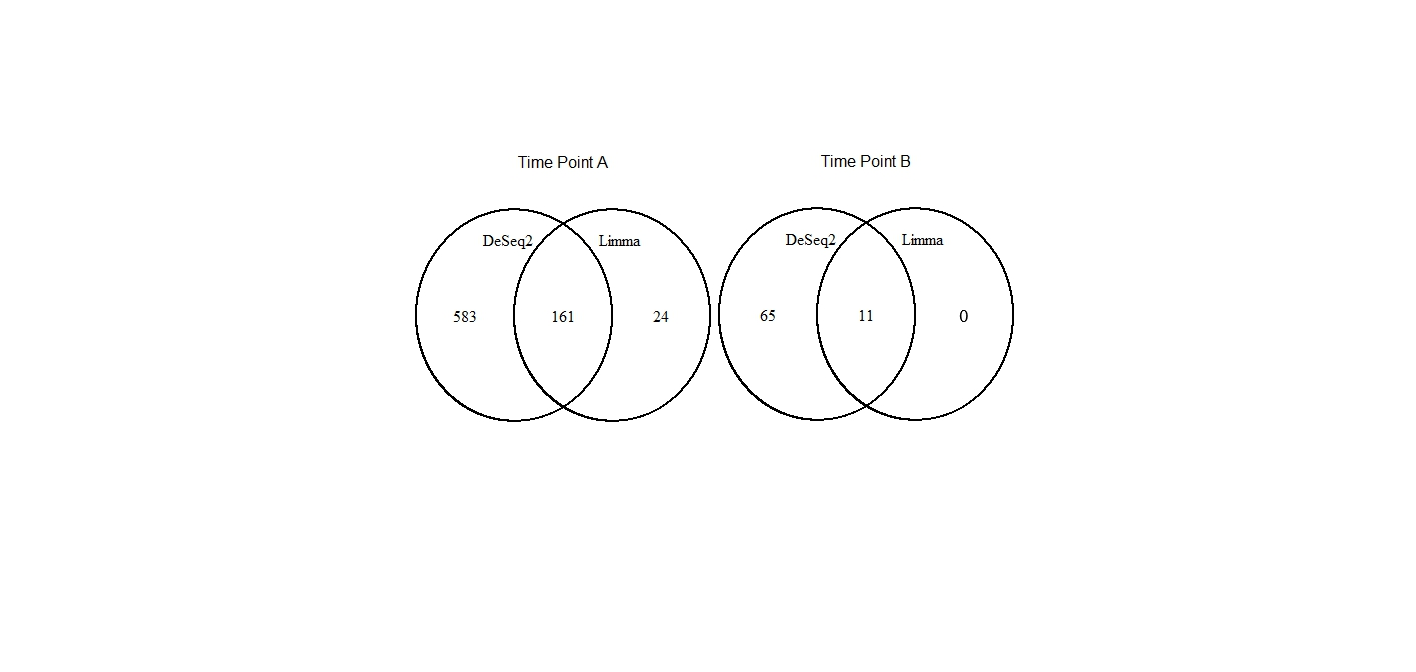


**Supplemental Figure 1. Comparison of statistical methods for RNAseq.** Venn diagram of differentially expressed genes (p<0.05) in the combined elevated [CO_2_], elevated temperature treatment comparing LIMMA and DESeq2 analyses for time points A and time point B.


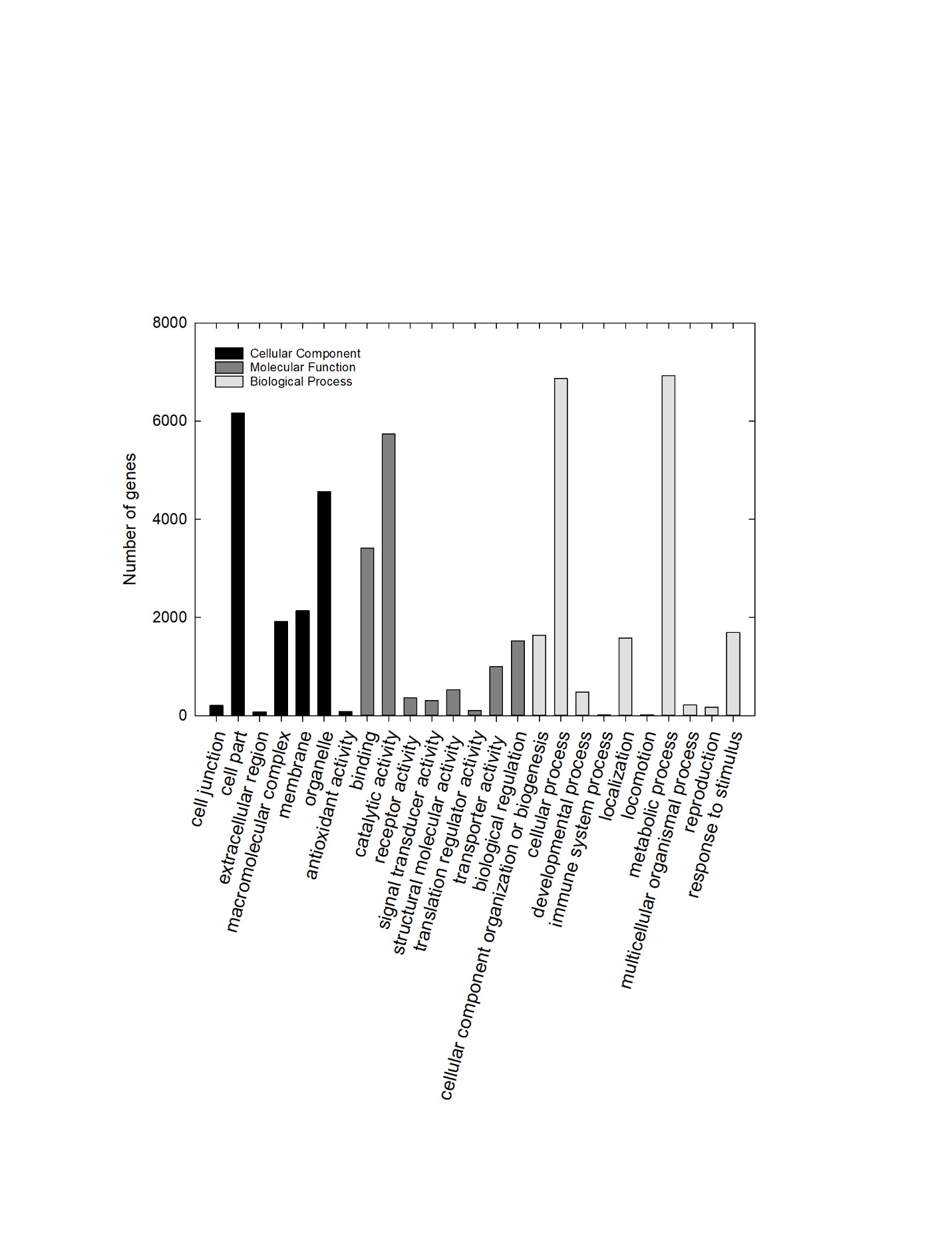


**Supplemental Figure 2: Gene ontology ‘slim’ classification of the *Panicum maximum* transcriptome.** The number of unigenes assigned to each GO slim terms in the *P. maximum* transcriptome is shown in the bar chart. Transcripts were classified into three categories: cellular components, molecular functions and biological processes. 11,538 total classified unigenes. False discovery rate of 0.05 was applied and categories with less than 10 genes were removed.


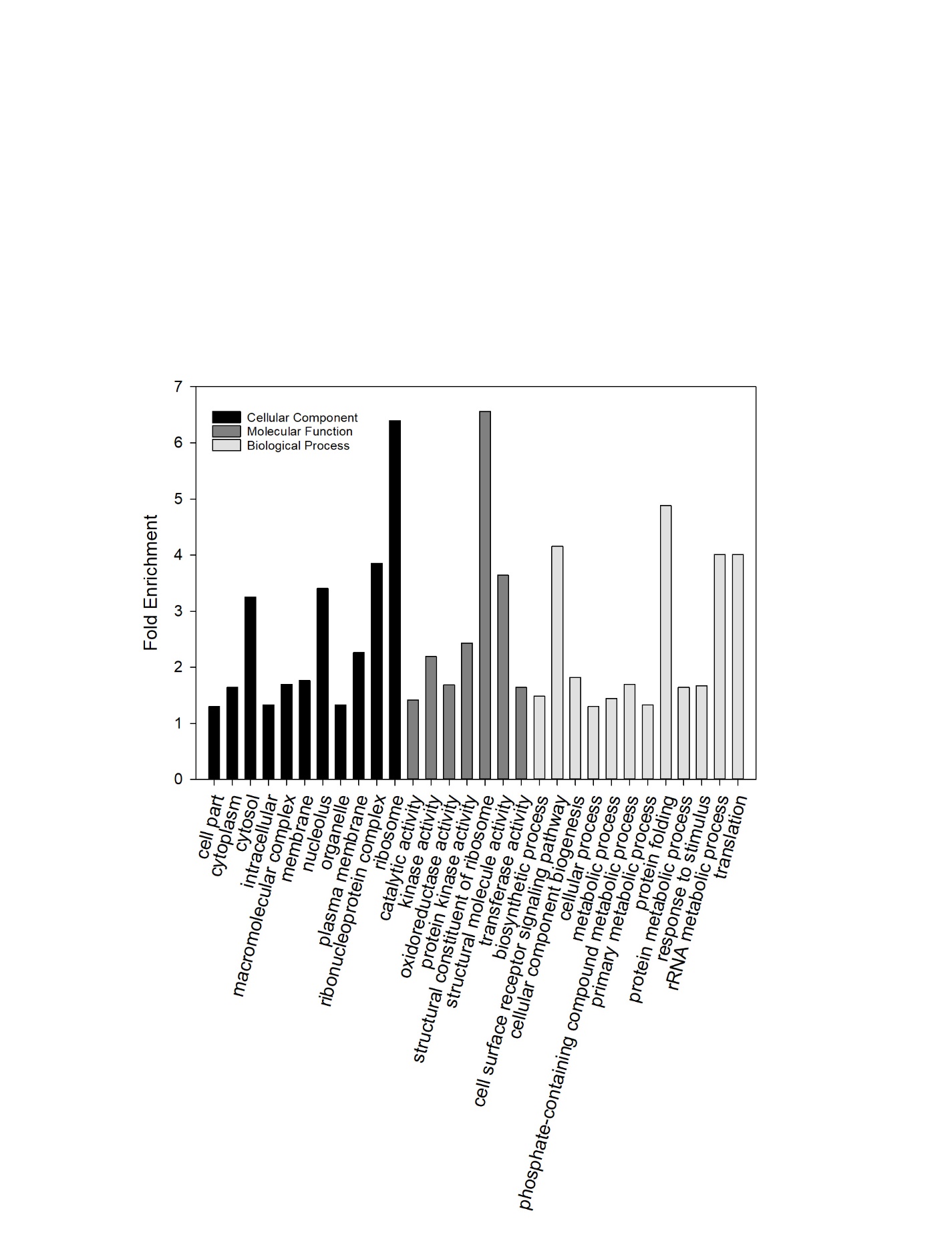


**Supplemental Figure 3: Gene ontology ‘slim’ classification of differently expressed genes (DEG) in plants grown in the combined elevated [CO_2_], elevated temperature treatment in time point A.** The average fold enrichment for groups of transcripts assigned to each GO slim term is shown. Transcripts were classified into three categories: cellular components, molecular functions and biological processes. 666 total genes were classified. A false discovery rate of 0.05 was applied.

**
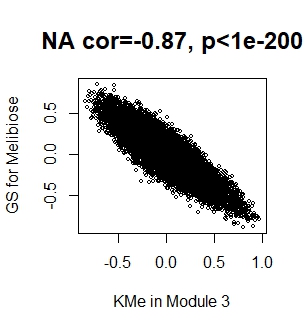
**

**Supplemental Figure 4:** Correlation between module membership in Module 3 and gene significance (GS) for melibiose. Eigengen based connectivity of module membership measure is plotted on the x axis (KMe). Correlation coefficient -0.87 and p<0.0001.


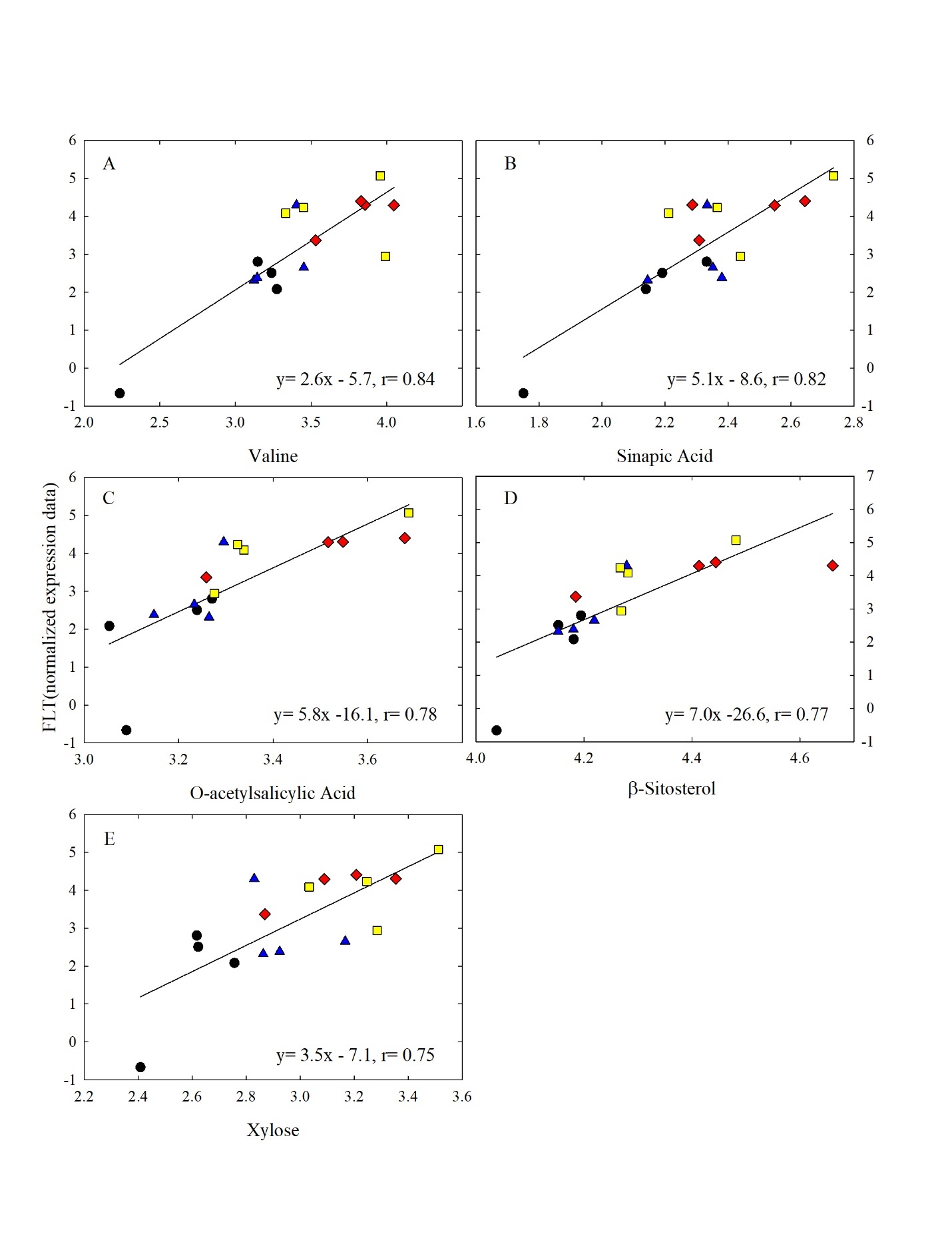


**Supplemental Figure 5: Linear correlations, from Time Point B, with Flowering Locus T and metabolite content.**  ‘At1g65480’ FLT (normalized expression level) correlation with metabolite [Log_10_ (relative concentration/ 100mg DW)] (A) Valine, (B) Sinapic acid, (C) O-acetylsalicylic acid, (D) β- Sitosterol, (E) Xylose. *Ambient (black, circle), elevated [CO_2_] (blue, triangle), elevated temperature (red, diamond), combination (yellow, square).*  *Linear regression equation and correlation coefficient are denoted on each panel.*


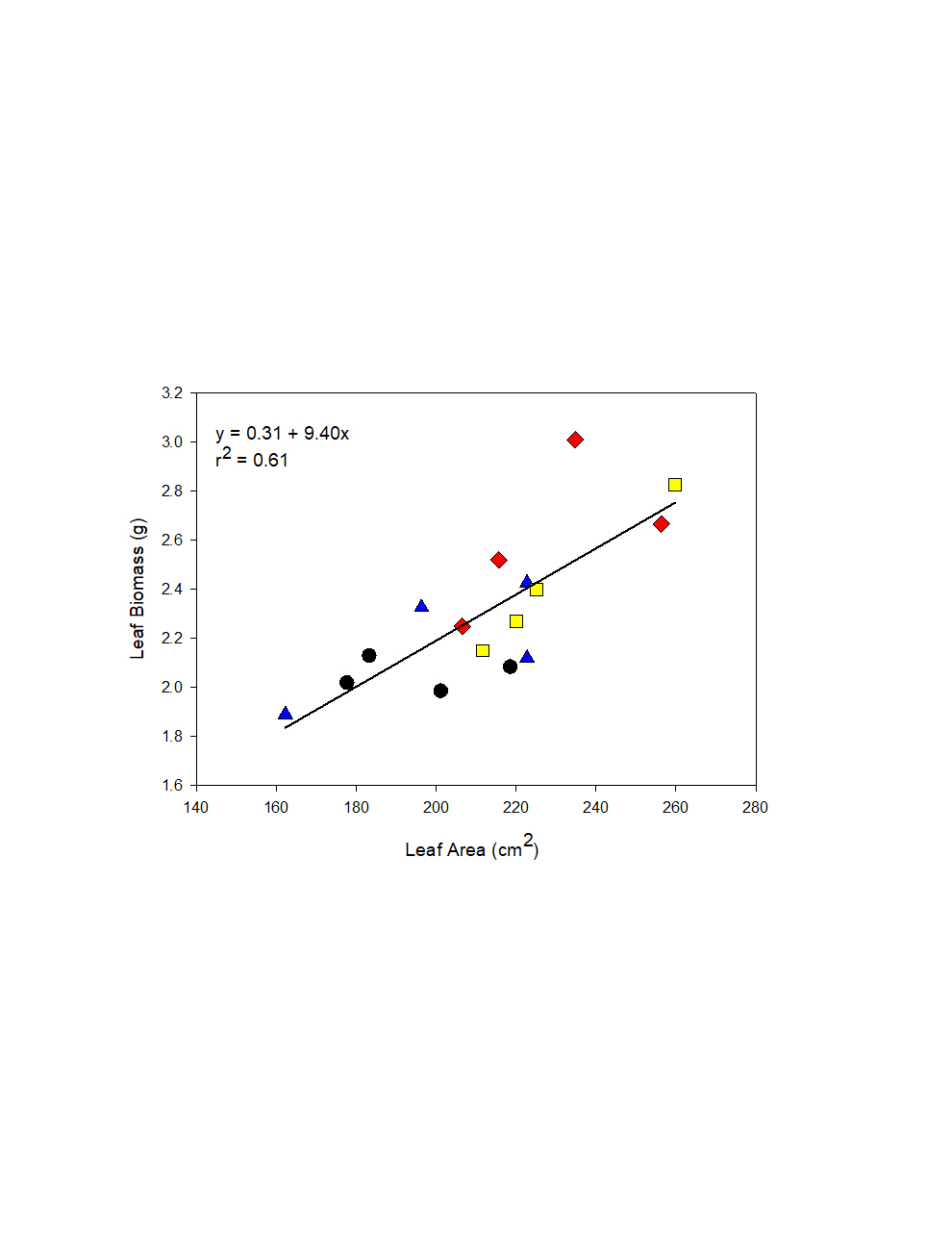


**Supplemental Figure 6: Relationship between individual leaf biomass and leaf area.** The linear correlation of Guinea grass individual leaf area and leaf biomass measured on Aug 22-29, 2014. *Ambient [CO_2_] and temperature (black, circle), Elevated [CO_2_], ambient temperature (blue, triangle), Ambient [CO_2_], Elevated temperature (red, diamond), Elevated [CO_2_] and temperature (yellow, square).*
